# Characterization of Computed Tomography Colonography Findings of Ulcerative Colitis-Associated Neoplasia

**DOI:** 10.1093/ibd/izaf303

**Published:** 2025-12-09

**Authors:** Yuta Kaieda, Shinya Sugimoto, Tatsuya Suzuki, Shunsuke Matsumoto, Hiroki Kiyohara, Kaoru Takabayashi, Yusuke Yoshimatsu, Koji Okabayashi, Kohei Shigeta, Ryoya Sakakibara, Yusuke Wakisaka, Soichiro Murakami, Masahiro Jinzaki, Yasushi Iwao, Yohei Mikami, Takanori Kanai

**Affiliations:** Division of Gastroenterology and Hepatology, Department of Internal Medicine, Keio University School of Medicine, Tokyo 160-8582, Japan; Division of Gastroenterology and Hepatology, Department of Internal Medicine, Keio University School of Medicine, Tokyo 160-8582, Japan; Department of Radiology, Keio University School of Medicine, Tokyo 160-8582, Japan; Department of Radiology, Keio University School of Medicine, Tokyo 160-8582, Japan; Division of Gastroenterology and Hepatology, Department of Internal Medicine, Keio University School of Medicine, Tokyo 160-8582, Japan; Center for Diagnostic and Therapeutic Endoscopy, Keio University School of Medicine, Tokyo 160-8582, Japan; Division of Gastroenterology and Hepatology, Department of Internal Medicine, Keio University School of Medicine, Tokyo 160-8582, Japan; Department of Surgery, Keio University School of Medicine, Tokyo 160-8582, Japan; Department of Surgery, Keio University School of Medicine, Tokyo 160-8582, Japan; Division of Gastroenterology and Hepatology, Department of Internal Medicine, Keio University School of Medicine, Tokyo 160-8582, Japan; Division of Gastroenterology and Hepatology, Department of Internal Medicine, Keio University School of Medicine, Tokyo 160-8582, Japan; Division of Gastroenterology and Hepatology, Department of Internal Medicine, Keio University School of Medicine, Tokyo 160-8582, Japan; Department of Radiology, Keio University School of Medicine, Tokyo 160-8582, Japan; Center for Preventive Medicine, Keio University, Tokyo 106-0041, Japan; Division of Gastroenterology and Hepatology, Department of Internal Medicine, Keio University School of Medicine, Tokyo 160-8582, Japan; Division of Gastroenterology and Hepatology, Department of Internal Medicine, Keio University School of Medicine, Tokyo 160-8582, Japan

**Keywords:** colitis-associated neoplasms, dysplasia, colorectal cancer, surveillance

## Abstract

**Background:**

Computed tomography colonography (CTC) is increasingly utilized for the evaluation of colorectal neoplasms. However, in patients with ulcerative colitis (UC), current European Crohn’s and Colitis Organisation guidelines recommend CTC only for limited indications, such as the presence of strictures.

**Objective**

This study aimed to assess the potential clinical utility of CTC by examining its detectability of UC-associated neoplasia (UCAN) lesions.

**Methods:**

This single-center, retrospective observational study included consecutive patients with UC who underwent preoperative CTC and were scheduled for pancolectomy for UCAN between January 2014 and June 2024. Lesion detectability on CTC was assessed in comparison with endoscopic findings, histopathological tumor depth, and morphological characteristics. Multivariable logistic regression was performed to identify factors associated with detectability on CTC.

**Results:**

Among 50 patients with 71 histologically confirmed lesions, 49% (35/71) were detectable by CTC. Detection was highest in advanced cancer (100%, 7/7), sessile (80%, 4/5) and depressed (80%, 8/10) morphologies, and lower in non-polypoid types such as superficial elevated (58%, 14/24) and flat (8%, 2/25) lesions. Detection by depth was 29% (12/42) for intramucosal, 75% (9/12) for submucosal, 100% (5/5) for muscularis propria, 73% (8/11) for subserosa/adventitia, and 100% (1/1) for serosal lesions. Flat morphology (adjusted odds ratio [aOR], 0.06; 95% confidence interval [CI], 0.01-0.27) and intramucosal invasion (aOR, 0.10; 95% CI, 0.02-0.46) were independently associated with non-detection.

**Conclusions and Relevance:**

Despite preoperative awareness of UCAN, CTC demonstrated limited sensitivity. While CTC may serve a complementary role in selected cases, endoscopy remains essential for comprehensive lesion detection.

Key Messages **What is already known?**

- Computed tomography colonography (CTC) is increasingly utilized for colorectal cancer screening.
- Data regarding the utility of CTC in detecting UC-associated neoplasia (UCAN) are limited.

**What is new here?**

- Detection rates of UCAN remained low despite prior knowledge of lesion.
- Lesions with subserosal invasion may exhibit solitary sclerotic changes without appreciable elevation—features not typically associated with sporadic neoplasia.
- Flat morphology and intramucosal lesions significantly predict UCAN invisibility on CTC.

**How can this study help patient care?**

- Our findings highlight important limitations of CTC in the UC surveillance context and reinforce the continued central role of endoscopy.

## Introduction

Ulcerative colitis (UC) is a chronic inflammatory condition characterized by immune-mediated inflammation of the colonic mucosa. Despite advances in medical management, no curative therapy has been established, and current treatment strategies focus on achieving and maintaining endoscopic remission. Patients with UC are known to have an elevated risk of developing colorectal cancer (CRC) compared with the general population.^1^ UC-associated neoplasia (UCAN) arises through an inflammation-driven, p53-mediated carcinogenic pathway and often exhibits endoscopic morphologies distinct from those of sporadic neoplasia (SN).^2^ These lesions are frequently non-polypoid and present in diverse forms that may be confounded by chronic mucosal inflammation, complicating their detection during colonoscopic surveillance.^3^^,^^4^ As the number of patients with long-standing UC increases—especially those benefiting from therapeutic advances that allow for colectomy avoidance—the demand for reliable CRC surveillance has grown.^5^

Computed tomography colonography (CTC) has emerged as a non-invasive alternative to conventional colonoscopy in CRC screening, particularly for visualizing the colon in cases of proximal strictures or incomplete endoscopic examination.^6^^,^^7^ However, its utility in UC has not been thoroughly investigated.^8^ Current European Crohn’s and Colitis Organisation (ECCO) guidelines recommend CTC only when endoscopic access is precluded by stenosis, primarily based on its role in evaluating inflammation rather than neoplasia.^9^ Moreover, existing evidence regarding CTC’s performance in detecting UCAN is limited to a small number of case reports.^8^^,^^10^^,^^11^ Given that SN typically presents as polypoid lesions while UCAN is often flat or superficially elevated,^12^ the diagnostic sensitivity of CTC for UCAN remains uncertain. Nonetheless, with a growing population of aging UC patients, some of whom may undergo CTC in clinical practice, clarifying its diagnostic utility is essential. In this study, we aimed to evaluate how UCAN is visualized on preoperative CTC by comparing imaging findings with those from endoscopy and resected pathologic specimens.

## Methods

### Patient Selection and Data Collection

This retrospective observational study was conducted at Keio University Hospital (Tokyo, Japan). Consecutive patients with a confirmed diagnosis of UC who underwent preoperative CTC prior to scheduled pancolectomy for UCAN between January 2014 and June 2024 were eligible for inclusion. Patients who underwent additional surgical intervention following endoscopic resection of UCAN, as well as those deemed inoperable, were excluded from the analysis. The study protocol was reviewed and approved by the Ethics Committee of Keio University School of Medicine (Approval No. 20150100).

### CTC Preparation and Procedure

Morphologic evaluation of UCAN was performed using CTC images, acquired as part of the routine preoperative evaluation to localize lesions and assess colonic and vascular anatomy. Bowel preparation included administration of 10 mL sodium picosulfate hydrate the night before the procedure, followed by 2 L of polyethylene glycol solution (Niflec EA Pharma Co., Ltd, Tokyo, Japan) and 40 mg of mosapride citrate hydrate on the morning of the examination. The CTC scan was performed in the afternoon.

A disposable rectal catheter (PROTOCO_2_L™ catheter set) was inserted, and the balloon was inflated immediately prior to imaging. Patients were sequentially positioned in left lateral, supine, and prone postures while carbon dioxide gas was insufflated at a pressure of 18-20 mmHg using the PROTOCO_2_L™ automatic insufflation system (EIDIA, Tokyo, Japan). CT imaging was performed using a 64-row multidetector scanner (Aquilion™ 64; Toshiba Medical Systems, Tokyo, Japan) from 2014 to 2015, and a 320-row scanner (Aquilion™ ONE; Canon Medical Systems, Tochigi, Japan) thereafter. Scans were obtained in both prone and supine positions to facilitate redistribution of luminal fluid.^13^ Fecal tagging using oral contrast was not implemented.

Non-contrast-enhanced CT was acquired in the prone position, followed by contrast-enhanced dynamic CT in the supine position. A nonionic contrast agent (300 mg I/mL) was administered at 2.0 mL/kg body weight via mechanical power injector over 28 seconds. Arterial-phase images were captured 15 seconds after a region of interest placed within the abdominal aorta at the level of the second to third lumbar vertebrae (L2-L3) reached 150 Hounsfield units (HU), as determined by bolus tracking. Venous-phase imaging commenced 60 seconds after contrast administration. Image acquisition parameters included a slice thickness and reconstruction interval of 0.5 mm.

### CTC Interpretation

Image analysis was conducted using a dedicated 3-dimensional imaging workstation (ZIOstation2™; Ziosoft, Tokyo, Japan). Routine interpretation of CTC images incorporated multiple reconstructed views, including air enema images, virtual endoscopic images, virtual endoscopy combined with multi-planar reconstruction (MPR), and virtual gross pathology projections. Image interpretation was independently performed by 2 experienced radiologists (T.S. and S.M.), each board-certified by the Japan Radiological Society, with a minimum of 1000 prior CTC readings and full responsibility for CTC interpretation at our institution throughout the study period. The radiologists conducted their evaluations within several days of image acquisition. Interpretations were performed independently and without direct consultation with the attending endoscopists or surgeons. However, relevant clinical information and endoscopic images were accessible as part of the preoperative workup for UCAN. Aware of the presence of lesions, the radiologists assessed whether the lesions were identifiable on CTC. All readings were subsequently reviewed and validated by 2 expert endoscopists (Y.K. and S.S.), both specializing in inflammatory bowel disease, through retrospective comparison with endoscopic images.

### Histopathologic Evaluation

Histopathological diagnosis was based on a comprehensive examination of the entire resected specimen by at least 2 experienced gastrointestinal pathologists. Diagnostic confirmation relied on standard hematoxylin and eosin staining, supplemented by immunohistochemical analysis of p53 and Ki67 expression, in accordance with previously established protocols.^14^^,^^15^ For this study, data were derived from a previously published cohort of patients diagnosed using these validated criteria.^2^^,^^5^^,^^12^^,^^16^ The cohort comprised cases of UC-associated high-grade dysplasia or adenocarcinoma and excluded non-dysplastic lesions, neuroendocrine tumors, sporadic adenoma or carcinomas, serrated lesions, traditional serrated adenomas, lesions of indefinite neoplastic potential, and UC-associated low-grade dysplasia.

### Endoscopic Evaluation

Endoscopic images, previously acquired and reported in earlier studies,^2^^,^^17^ were retrospectively compared with corresponding CTC images. Briefly, neoplastic lesions were initially identified via targeted biopsy and subsequently examined in detail using high-resolution endoscopes (PCF-H290I, PCF-Q260AI, CF-H290I, GIF-XZ1200, PCF-H290ZI, or CF-HQ290I [Olympus, Tokyo, Japan]; or EC-L600ZP7 [Fujifilm, Tokyo, Japan]). Lesions were visualized with chromoendoscopy using 0.1%-0.2% indigo carmine dye.^2^^,^^17^ Based on the SCENIC consensus statement, lesions presumed to involve the intramucosa or submucosa were morphologically classified as pedunculated, sessile, superficial elevated, flat, or depressed.^18^ Tumor morphology was determined macroscopically based on the preoperative endoscopic appearance and did not rely on postoperative histopathologic findings.^19^ The severity of mucosal inflammation was assessed using the Mayo endoscopic subscore (MES).^20^

### Statistical Analysis

Continuous variables are expressed as median values with interquartile ranges (IQR), while categorical variables are presented as percentages. The Wilcoxon rank-sum test was used for comparisons of continuous variables, and Fisher’s exact test was applied for intergroup comparisons of categorical variables. Logistic regression analyses were conducted to identify factors associated with CTC-based detection of UCAN. Both univariable and multivariable models were constructed. Given the constraint on the number of covariates per model, 2 multivariable models were developed: Model 1 assessed the association of MES and morphologic features with CTC detectability, while Model 2 examined MES and histopathologic tumor depth. All statistical analyses were performed using R Studio software version 4.3.2, with 2-sided *P*-values < .05 considered statistically significant.

## Results

### Patient Profile

**Figure 1.**
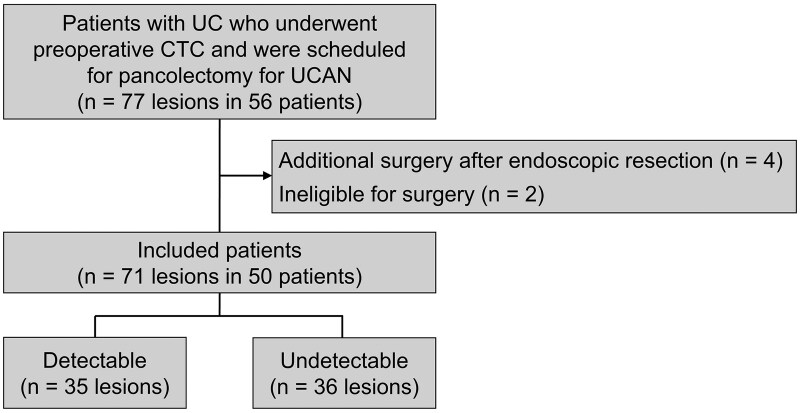
Study flow diagram. Among 56 patients who underwent CTC prior to pancolectomy, 50 were included in the final analysis. A total of 35 lesions were detectable on CTC imaging. UCAN, ulcerative colitis-associated neoplasia; CTC, computed tomography colonography.

Between January 2014 and June 2024, a total of 56 consecutive patients with UC underwent CTC as part of the preoperative evaluation for pancolectomy performed for UCAN at our institution. Of these, 6 patients were excluded: 4 had undergone additional surgical resection following prior endoscopic resection of UCAN, and 2 were deemed inoperable. Consequently, 50 patients (28 men and 22 women) with a cumulative total of 71 histologically confirmed lesions were included in the final analysis (Figure 1). At the time of CTC, the median patient age was 51 years (IQR, 43-66), and the median disease duration was 19 years (IQR, 13-26). Notably, nearly 90% of patients were in endoscopic remission, as defined by a MES of 0 or 1 (Table 1). All but the first 3 patients were examined using a 320-row scanner rather than a 64-row scanner, and differences in detection rate attributable to scanner resolution could not be evaluated.

**Table 1.** Characteristics of lesions included in this study.

	**All ** **(*n* = 71)**	**Detectable** ** (*n* = 35)**	**Undetectable** ** (*n* = 36)**	*P*
**Age, median (IQR)**	51 (43-66)	48 (40-67)	53.5 (43-66)	.534
**Duration of disease, median (IQR)**	19 (13-26)	20 (11-28)	18 (13-24)	.872
**Sex (male/female), *n* (%)**	33 (46.5%)/38 (53.5%)	15 (42.9%)/20 (57.1%)	18 (50.0%)/18 (50.0%)	.637
**Extent of disease, *n* (%)**				.055
** Pancolitis**	64 (90.2%)	29 (82.9%)	35 (97.2%)	
** Left-sided colitis**	7 (9.8%)	6 (17.1%)	1 (2.8%)	
** Proctitis**	0	0	0	
**Mayo endoscopic subscore, *n* (%)**				.021
** 0**	48 (67.6%)	18 (51.4%)	30 (83.3%)	
** 1**	16 (22.5%)	11 (31.4%)	5 (13.9%)	
** 2**	5 (7.1%)	4 (11.4%)	1 (2.8%)	
** 3**	2 (2.8%)	2 (5.7%)	0	
**Size** ^a^ **, mm**	30 (15-47)	30 (15.75-50)	20 (9.5-40)	.169
**Lesion location, *n* (%)**				.943
** Rectum**	31 (43.7%)	16 (45.7%)	15 (41.7%)	
** Sigmoid colon**	24 (33.8%)	11 (31.4%)	13 (36.1%)	
** Descending colon**	3 (4.2%)	1 (2.9%)	2 (5.6%)	
** Transverse colon**	8 (11.3%)	4 (11.4%)	4 (11.1%)	
** Ascending colon**	4 (5.6%)	2 (5.7%)	2 (5.6%)	
** Cecum**	1 (1.4%)	1 (2.9%)	0	
**Morphology, *n* (%)**				<.001
** Sessile**	5 (7.1%)	4 (11.4%)	1 (2.8%)	
** Superficial elevated**	24 (33.8%)	14 (40.0%)	10 (27.8%)	
** Flat**	25 (35.2%)	2 (5.7%)	23 (63.9%)	
** Depressed**	10 (14.1%)	8 (22.9%)	2 (5.6%)	
** Type1-5**	7 (9.8%)	7 (20.0%)	0	
**Depth, *n* (%)**				<.001
** Intramucosa (M, Tis)**	42 (59.1%)	12 (34.3%)	30 (83.3%)	
** Submucosa (SM, T1)**	12 (16.9%)	9 (25.7%)	3 (8.3%)	
** Muscularis propria (MP, T2)**	5 (7.1%)	5 (14.3%)	0	
**Subserosa or adventitia (SS or A, T3)**	11 (15.5%)	8 (22.9%)	3 (8.3%)	
** Serosa (SE, T4)**	1 (1.4%)	1 (2.9%)	0	

a Four lesions with missing data were excluded from the analysis.

### Lesion Profile

Among the 71 neoplastic lesions, 35 (49.3%) were detectable by CTC. The median interval between colonoscopy and CTC was 32 days (range, 0-48). No serious CTC-related complications, such as perforation or bleeding, were reported. Lesion characteristics are summarized in Table 1. Consistent with previous findings,^2^ nearly 80% of lesions were located in the rectum (43.7%) and sigmoid colon (33.8%). Based on endoscopic morphology, lesions were classified as sessile (7.1%, *n* = 5), superficial elevated (33.8%, *n* = 24), flat (35.2%, *n* = 25), depressed (14.1%, *n* = 10), or advanced cancer type (9.8%, *n* = 7). The median lesion size was 30 mm. Histopathologically, the majority of lesions were early-stage: 59.1% were intramucosal and 16.9% invaded the submucosa. No additional lesions were detected by CTC that had not already been identified on colonoscopy. However, pathological examination of the colectomy specimens revealed 4 additional intramucosal lesions that were not detected by either colonoscopy or CTC.

### CTC Detection by Lesion Location and Morphology

**Figure 2.**
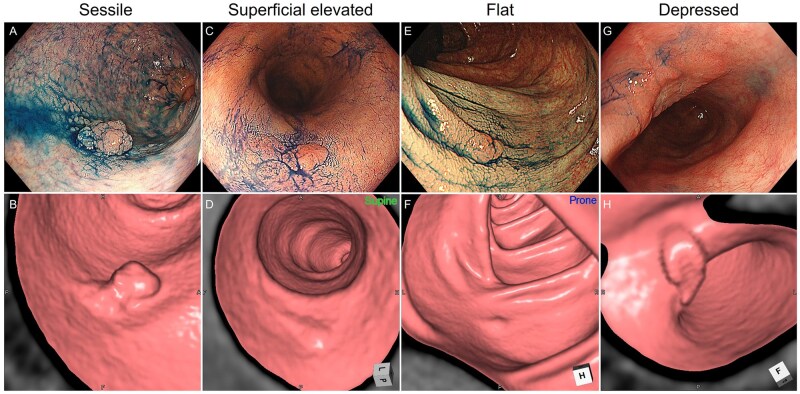
Representative lesions detected on initial CTC interpretation. Lesions are categorized by SCENIC classification: (A, B) sessile type; (C, D) superficial elevated type; (E, F) flat type; and (G, H) depressed type.

**Figure 3.**
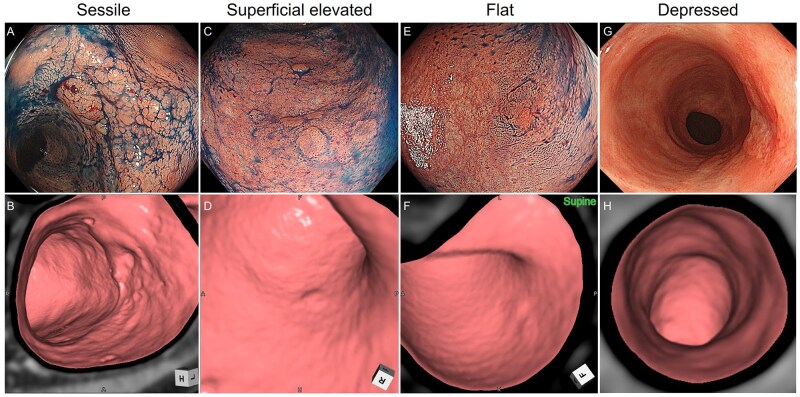
Representative lesions not detected on initial CTC interpretation. CTC images are shown in reference to lesion locations identified by colonoscopy.

**Figure 4.**
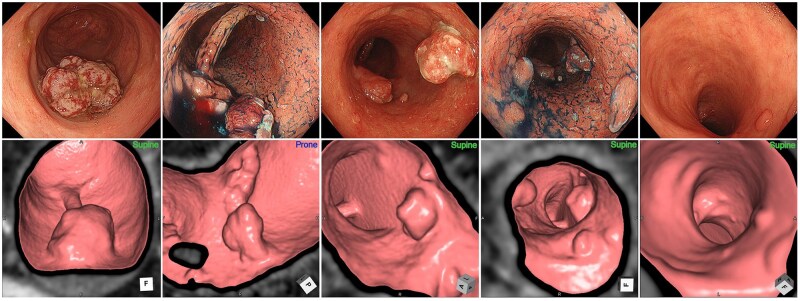
Representative images of inflammatory polyps.

Detection rates by lesion location were as follows: rectum, 51.6% (16/31); sigmoid colon, 45.8% (11/24); descending colon, 33.3% (1/3); and right-sided colon, 53.8% (7/13) (Table 1). We next analyzed CTC detectability according to endoscopic morphological classification (Figures 2A-H and 3A-H). Detection rates were highest among advanced cancer type lesions (100%, 7/7), sessile lesions (80.0%, 4/5), and depressed lesions (80.0%, 8/10). In contrast, among non-polypoid lesions, detection was lower: 58.3% (14/24) for superficial elevated and only 8.0% (2/25) for flat lesions. These findings align with prior reports in non-UC populations,^21^ where flat (0-IIb) lesions are also notably difficult to detect using CTC. In rare cases, detection was possible when atypical features were present, such as submucosal tumor-like growths that appeared as slightly elevated regions blending into the surrounding mucosa, later confirmed histopathologically as 0-IIb (Figure 2E and F). Although not a primary focus of this study, inflammatory polyps visualized on endoscopy were also identified as elevated lesions on CTC, complicating qualitative differentiation without supplementary endoscopic information (Figure 4).

### CTC Detection by Tumor Depth

**Figure 5.**
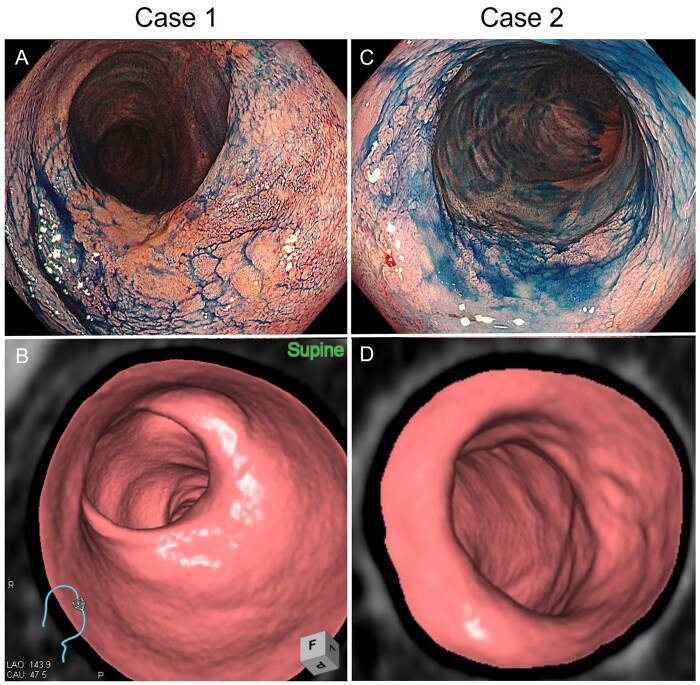
Representative images of undetectable lesions with advanced subserosal invasion. The 2 lesions presented appeared as solitary sclerotic areas without elevation. Case 3 corresponds to the lesion shown in Figures 3G and 3H and is not repeated here.

We next examined the association between histopathologic tumor depth and CTC detectability. Among the 71 lesions, detection rates stratified by depth of invasion were as follows: 28.6% (12/42) for intramucosal (M), 75.0% (9/12) for submucosal (SM), 100% (5/5) for muscularis propria (MP), 72.7% (8/11) for subserosa (SS) or adventitia (A), and 100% (1/1) for serosal (SE) invasion. Although the low detection rate for early-stage intramucosal tumors was anticipated, we noted the unexpectedly modest detection rate for more advanced SS lesions. Upon reviewing the corresponding endoscopic images of the 3 SS lesions that were not detected on initial CTC interpretation, all were morphologically classified according to SCENIC criteria (Figures 3G and 5) and did not exhibit the prototypical advanced cancer appearance. Retrospective image comparison revealed that these lesions were associated with asymmetric, sclerotic wall thickening on CTC, suggesting a unique invasion pattern of UCAN that differs from that observed in SN.

### Factors Contributing to Lesion Detection

To identify clinical and pathological variables associated with CTC detectability, we conducted univariable and multivariable logistic regression analyses (Table 2). In univariable analysis, flat morphology was strongly associated with reduced odds of detection (odds ratio [OR], 0.06; 95% confidence interval [CI], 0.01-0.27; *P *= .001), as was intramucosal invasion depth (OR, 0.13; 95% CI, 0.03-0.53; *P *= .007). Additionally, MES 0 or 1 showed a trend toward lower detection (OR, 0.14; 95% CI, 0.01-0.87; *P *= .074), indicating its potential relevance as a contributing factor. Notably, of the 25 flat lesions, 23 (92.0%) were classified as M and only 2 (8.0%) as SM, indicating that flat morphology and mucosal confinement were not statistically independent. To address potential multicollinearity, we constructed 2 separate multivariable models. Model 1 included MES and morphology, and identified flat morphology as a significant independent predictor of non-detection (adjusted OR, 0.06; 95% CI, 0.01-0.27; *P *= .001). Model 2 included MES and depth of invasion, showing that mucosal invasion independently predicted undetectability (adjusted OR, 0.10; 95% CI, 0.02-0.46; *P *= .004). These findings collectively indicate that flat morphology and shallow invasion depth (limited to the mucosa) are significant independent predictors of UCAN undetectability on CTC.

**Table 2.** Univariable and multivariable logistic regression analyses of factors associated with lesion detection on CTC.

	Univariable analysis			Multivariable analysis Model 1			Multivariable analysis Model 2		
Predictor factor	Odds ratio	95% CI	*P*-value	Odds ratio	95% CI	*P*-value	Odds ratio	95% CI	*P*-value
**Age ≧ 50 y.o.**	0.93	0.73-1.18	.558						
**Duration ≧ 20years**	1.20	0.95-1.53	.126						
**Male**	0.75	0.29-1.91	.547						
**Pancolitis**	Reference								
**Left-sided colitis**	7.24	1.15-140	.074						
**Mayo endoscopic subscore: 0, 1**	0.14	0.01-0.87	.074	0.13	0.004-1.33	.137	0.16	0.01-1.50	.134
**Mayo endoscopic subscore: 2, 3**	Reference			Reference			Reference		
**Size: <10 mm**	0.26	0.03-1.28	.122						
**Size: 10-20 mm**	0.77	0.26-2.29	.640						
**Size: >20 mm**	Reference								
**Location of lesion: Rectum**	Reference								
**Location of lesion: Sigmoid colon**	0.79	0.27-2.31	.671						
**Location of lesion: Descending colon**	0.47	0.02-5.39	.553						
**Location of lesion: Right-sided**	1.09	0.30-4.12	.892						
**Morphology: Superficial elevated**	Reference			Reference					
**Morphology: Sessile**	2.86	0.35-60.5	.379	1.89	0.19-42.5	.613			
**Morphology: Flat**	0.06	0.01-0.27	.001	0.06	0.01-0.27	.001			
**Morphology: Depressed**	2.86	0.56-21.7	.239	2.55	0.48-19.8	.305			
**Morphology: Advanced (Type 1-5)**	N.A.^a^	–	–	N.A.^a^	–	–			
**Depth: Submucosa (SM, T1)**	Reference						Reference		
**Depth: Intramucosa (M, Tis)**	0.13	0.03-0.53	.007				0.10	0.02-0.46	.004
**Depth: Muscularis propria (MP, T2)**	N.A.^a^	–	–				N.A.^a^	–	–
**Depth: Subserosa or adventitia (SS or A, T3)**	0.89	0.13-6.06	.901				0.80	0.12-5.51	.816
**Depth: Serosa (SE, T4)**	N.A.^a^	–	–				N.A.^a^	–	–

a Odds ratios could not be calculated because of absence of events in one or more comparison groups.

## Discussion

To our knowledge, this is the first study to systematically evaluate the detectability of UCAN using CTC. Despite being interpreted with prior knowledge of the presence of neoplastic lesions, CTC failed to detect approximately half of the UCAN lesions included in this study. Multivariable analysis identified flat morphology and confinement of tumor invasion to the mucosa as independent predictors of non-detectability. While endoscopy remains the gold standard for colorectal neoplasia surveillance in UC, the growing use of CTC and the aging population of UC patients may increasingly necessitate interpretation of CTC images in this context. Accordingly, familiarity with UCAN-specific imaging characteristics is essential for radiologists tasked with evaluating these cases.

CTC has gained traction as a non-invasive screening tool for SN^7^^,^^22^; however, its application in patients with UC remains limited. This is likely because of concerns regarding the risk of intestinal perforation in inflamed bowel, coupled with the prevailing preference for colonoscopy, which enables direct evaluation of mucosal inflammation. Although CTC is contraindicated in cases of acute colitis,^23^ a study by White et al.^24^ demonstrated that CTC could be safely performed in patients with colitis, including those with inflammatory bowel disease, without any procedural complications.

Accurate identification of advanced cancer remains a critical clinical priority. In our cohort, 17 lesions were pathologically classified as advanced cancer, of which 14 (82.4%) were detectable by CTC. Notably, 3 lesions with SS were not recognized as neoplastic on CTC, even by radiologists highly experienced in SN interpretation. These lesions exhibited solitary sclerotic changes without appreciable elevation—features not typically associated with SN and therefore easily overlooked (Figure 5). This observation underscores the need for radiologists to recognize imaging patterns unique to UCAN, which may deviate substantially from those observed in sporadic cases. Such trainable characteristic findings may serve as imaging features that could be learned by future artificial intelligence–based detection systems or incorporated into focused education programs for radiologists to potentially increase the diagnostic yield of CTC.

CTC offers several advantages, including its operator-independent image acquisition and the ability to evaluate both intraluminal and extraluminal structures.^25^ It enables visualization of the mucosa beneath colonic folds, a region that may be inadequately examined during conventional colonoscopy. Furthermore, CTC may be particularly useful in elderly patients with multiple comorbidities, or in individuals with adhesions or strictures that preclude complete endoscopic evaluation. It also serves as a viable alternative for patients who are unwilling or unable to undergo colonoscopy. Nonetheless, colonoscopy continues to be prioritized in UC surveillance, not only for inflammatory assessment but also for neoplasia detection, owing to the predominance of flat morphologies in UCAN.^2^ In the present study, the detection rate of flat-type UCAN was only 8% (2/25), despite such lesions accounting for 35% (25/71) of all cases. This finding strongly suggests that CTC is insufficient for surveillance of flat-type UCAN and reinforces the indispensable role of endoscopy in this population.

This study has several limitations. First, it was a single-center, retrospective analysis with a relatively small sample size and lacked a control group, precluding the calculation of diagnostic performance metrics such as positive and negative predictive values or receiver operating characteristic (ROC) curves. Given that routine CTC screening for CRC in patients with UC is not currently practiced, our analysis was necessarily limited to retrospective evaluation of preoperative CTCs performed in patients with known UCAN. As this study included only patients with established UCAN, representing a population with the highest pretest probability, its findings may not be generalizable to patients without endoscopically detected lesions. Although the diagnostic performance of CTC in a surveillance population could not be evaluated, this investigation serves as an essential first step in characterizing CTC findings in the presence of UCAN. Future studies are needed to evaluate the generalizability of these findings to surveillance populations in which UCAN status is unknown. Second, qualitative assessment of polyps—particularly inflammatory polyps (Figure 4)—remains challenging in CTC. Although correlation with prior endoscopic findings may aid in distinguishing such lesions, definitive diagnosis of dysplasia still requires endoscopic and histopathologic confirmation. Because inflammatory polyps can mimic dysplastic lesions on imaging, their presence may complicate interpretation. Although inflammatory polyps themselves are not associated with the development of cancer,^26^ their presence can diminish the detectability of neoplastic lesions on CTC, limiting the potential improvement in cancer detection with this cross-sectional technique. Given that UCAN frequently includes lesions that are difficult to identify even with high-resolution endoscopy, direct comparison to findings from SN is inappropriate. This study represents a foundational effort to characterize UCAN on CTC and to compare morphological findings with those observed during endoscopy. Further studies are warranted to establish the clinical relevance and diagnostic role of CTC in this setting. Third, the apparently higher detection rate among patients with MES 2-3 likely reflects a selection bias. During active disease, flat UCAN lesions may be obscured by inflammation such as edema or ulceration, reducing their detectability by both endoscopy and CTC. However, lesions that remain visible despite inflammation often have more conspicuous imaging characteristics—such as elevation, depression, or deeper invasion—making them easier to detect on CTC. Therefore, this numerically higher detection rate should be interpreted with caution, as it likely represents selection bias rather improved diagnostic performance. Fourth, internal validation of CTC reading accuracy was limited by the lack of other radiologists at our institution with sufficient CTC interpretation expertise. Given that reading proficiency is a known confounding factor, interpretation bias was minimized by fixing the initial CTC assessments performed by 2 experienced radiologists in the clinical setting. Accuracy of lesion identification was subsequently verified by expert endoscopists. Nonetheless, external validation at additional centers with broader reader expertise is required to confirm these findings.

In conclusion, although CTC was able to detect many cases of UCAN, its diagnostic performance was markedly reduced for non-polypoid lesions. The finding that detection rates remained low even when radiologists reviewed images with prior knowledge of lesion presence highlights inherent limitations in CTC-based detection. While CTC may offer a practical alternative in cases where endoscopy is contraindicated or not feasible, its limited sensitivity for early-stage lesions and the potential for false-positive interpretations as a result of inflammatory polyps necessitate caution. Endoscopy remains indispensable for reliable surveillance of UCAN.

## Data Availability

The data underlying this article cannot be shared publicly due to ethical restrictions and privacy concerns. The data will be shared on reasonable request to the corresponding author.
